# Supplementary material for: Investigating the use of ultrasonography for the antenatal diagnosis of structural congenital anomalies in low-income and middle-income countries: systematic review protocol
Source: BMJ Paediatr Open. 2019 Sep 13;3(1):e000538. doi: 10.1136/bmjpo-2019-000538 (PMC6782034; doi:10.1136/bmjpo-2019-000538)
Supplement: Author's manuscript [file bmjpo-2019-000538.draft_revisions.pdf]

BMJ Paediatrics Open

BMJ Paediatrics Open is committed to open peer review. As part of this commitment we make the peer review history of every article we publish publicly available.

When an article is published we post the peer reviewers' comments and the authors' responses online. We also post the versions of the paper that were used during peer review. These are the versions that the peer review comments apply to.

The versions of the paper that follow are the versions that were submitted during the peer review process. They are not the versions of record or the final published versions. They should not be cited or distributed as the published version of this manuscript.

BMJ Paediatrics Open is an open access journal and the full, final, typeset and author-corrected version of record of the manuscript is available on our site with no access controls, subscription charges or pay-per-view fees (http://bmjpaedsopen.bmj.com).

If you have any questions on BMJ Paediatrics Open's open peer review process please email info.bmjpo@bmj.com

BMJ Paediatrics Open

Investigating the Use of Ultrasonography for the Antenatal Diagnosis of Structural Congenital Anomalies in Low- and Middle-Income Countries: A Systematic Review Protocol

Journal:	*BMJ Paediatrics Open*
Manuscript ID	bmjpo-2019-000538
Article Type:	Protocol
Date Submitted by the Author:	24-Jun-2019

Complete List of Authors:	Goley, Stephanie; King's College London, Department of Global Health & Social Medicine<br>Sakula-Barry, Sidonie; World Cancer Research Fund<br>Kelly, Ann; King's College London, Department of Global Health & Social Medicine<br>Wright, Naomi; King's College London, King's Centre for Global Health and Health Partnerships

Keywords:	Congenital Abnorm, Paediatric Surgery, Neonatology, Imaging

SCHOLARONE™
Manuscripts

Investigating the Use of Ultrasonography for the Antenatal Diagnosis of Structural Congenital Anomalies in Low- and Middle-Income Countries: A Systematic Review Protocol

Stephanie Goley[1], Sidonie Sakula-Barry[2], Ann Kelly[1], Naomi Wright[3]

June 2019

PROSPERO Registration: CRD42019105620
Registered on February 14, 2019
Prepared according to Prisma-P Guidelines

[1]Department of Global Health & Social Medicine, King's College London, London, UK
[2]World Cancer Research Fund UK, London, UK
[3]King's Centre for Global Health and Health Partnerships, School of Population Health and Environmental Sciences, King's College London, London, UK

Author Email Addresses:
info@stephgoley.com
sidonie.sakula-barry1@alumni.lshtm.ac.uk
ann.kelly@kcl.ac.uk
paedsurg.research@gmail.com

Corresponding Author:
Stephanie Goley, BA MSc
Department of Global Health & Social Medicine, King's College London, London, UK
Correspondence to: Centre for Global Health and Health Partnerships, Suite 2.13 Weston Education Centre, Cutcombe Road, London, SE5 9RJ
Telephone: +1 (815) 830-5360

Confidential: For Review Only

What is known?
• 97% of deaths from congenital anomalies occur in developing countries. Many of these deaths could be prevented with early diagnosis and intervention.<br>• Ultrasound machines are widely accessible and commonplace in HICs, but a number of factors limit the accessibility and effectiveness of ultrasound in LMICs.
**What will this study add?**
• To systematically investigate, for the first time, the availability and effectiveness of antenatal ultrasound in LMICs in order to elucidate disparities in congenital anomaly detection.<br>• To evaluate the effects of antenatal ultrasound on the morbidity and mortality rates of neonates with a structural congenital anomaly in LMICs.

ABSTRACT

Introduction: Congenital anomalies are the 5th leading cause of under-5 mortality globally. The greatest burden is faced by those in developing countries, where over 95% of deaths occur. Many of these deaths may have been preventable through antenatal diagnosis and early intervention. This study aims to conduct a systematic review that investigates the use of antenatal ultrasound to diagnose congenital anomalies and improve the health outcomes of infants in low- and middle-income countries (LMICs).

Methods and Analysis: A systematic literature review will be conducted using three search strings: 1) structural congenital anomalies; 2) LMICs; and 3) antenatal diagnosis. Four electronic databases will be searched: MEDLINE, EMBASE, PubMed, and the Cochrane Library. Title, abstract and full text screening will be undertaken in duplicate by two reviewers independently using Covidence. Consensus will be sought from the wider authorship for discrepancies. Data extraction will be undertaken by the principal investigator. The primary analysis will focus on the availability and effectiveness of antenatal ultrasound for structural congenital anomalies. Secondary outcomes will include neonatal morbidity and mortality, termination rates, and referral rates for further antenatal care. Descriptive statistics and a narrative synthesis will be included in the final report. Methodological quality of the included studies will be evaluated using the Cochrane-approved ROBINS-I and RoB 2.0 tools.

Ethics and Dissemination: Ethical approval is not required for conducting the systematic review as there will be no direct collection of data from individuals. The results will be submitted for publication in a scientific journal and presented internationally.

Conclusion: This is the first study, to our knowledge, to systematically review current literature on the use of antenatal ultrasound for the detection of congenital anomalies in LMICs. This is vital to define current practice, highlight global disparities and evaluate effects on health outcomes for infants in low-resource settings.

Confidential: For Review Only

INTRODUCTION

Congenital anomalies represent 9% of the global burden of surgical disease and are one of the leading causes of infant morbidity and mortality globally [1-4]. The burden of congenital anomalies is much greater in low- and middle-income countries (LMICs), comprising over 95% of deaths from these conditions [5, 6]. According to a report by the WHO, every year approximately 303,000 infants die within 4 weeks of birth due to congenital anomalies [7]. However, this is likely an underestimation, as statistics are undoubtedly skewed from underreporting, a lack of congenital anomaly registries, and unreliable medical records in many LMICs.

Structural congenital anomalies are physical deformities that occur during intrauterine development [7]. These anomalies include, but are not limited to, gastro-intestinal malformations, cleft lip and palate, heart defects, musculoskeletal anomalies, and neural tube defects [8]. If detected early and provided appropriate surgical intervention following birth, many structural congenital anomalies can be corrected avoiding preventable death or disability [7].

Advancements in ultrasound technology have allowed for early detection of structural congenital anomalies, which allows mothers and physicians to plan for an appropriate place of delivery and surgical intervention after birth [9]. In many cases, this can result in a significantly reduced risk of morbidity and mortality [10]. While ultrasound machines are widely accessible and commonplace in high-income countries, a number of factors limit the access and effectiveness of antenatal ultrasound in LMICs.

This study aims to systematically investigate, for the first time, the availability and effectiveness of antenatal ultrasound in the diagnosis of structural congenital anomalies in LMICs. Furthermore it aims to evaluate the effects of antenatal ultrasound on mortality and morbidity for neonates with a structural congenital anomaly in LMICs. Such information is vital to help clarify the existing disparities in antenatal ultrasound provision and the potential benefits for improved health outcomes through the delivery of this service in low-resource settings.

METHODS

PRISMA-P guidelines will be followed in conducting this systematic review [11, 12] (Supplementary File 1). If there are amendments to the protocol, they will be reported in the publication of the results.

Aim

To conduct a systematic review that investigates the use of antenatal ultrasound to diagnose congenital anomalies and improve the health outcomes of infants in low- and middle-income countries.

Confidential: For Review Only

Objectives

1. To systematically catalog studies that focus on the antenatal diagnosis of structural congenital anomalies in LMICs.
2. To evaluate the utilization and effectiveness of ultrasound machines in the antenatal diagnosis of structural congenital anomalies and to report the current practices and policies regarding congenital anomaly detection in LMICs.
3. To evaluate the effects of antenatal ultrasound on the morbidity and mortality rates of neonates with a structural congenital anomaly in LMICs.
4. To critique the methodological quality of the included articles.

Patient and Public Involvement

Given that this is a systematic literature review, there will be no patient or public involvement. However, all findings will be appropriately disseminated to the public.

Search Strategy

A search will be conducted using three search strings (Table 1). Search string 1 will encompass structural congenital anomalies. Search string 2 will focus on the setting - LMICs. Finally, search string 3 will look at the antenatal diagnosis of structural congenital anomalies, particularly focusing on the use of ultrasound machines for detection. Borlean operators 'and' and 'or' will be utilized within the search to combine the search terms.

Table 1: Search Strings

Search String 1	Search String 2	Search String 3
Congenital Anomalies, Congenital Abnormalities, Congenital Malformation, Fetal Malformation, Birth Defects, Anencephaly, Conjoined Twins, Congenital Heart Defects, Anorectal Malformations, Anal Stenosis, Anal Atresia, Imperforate Anus, Biliary Atresia, Choledochal Cyst, Diaphragmatic Eventration, Esophageal Atresia,	LMICs, Low- and Middle-Income Countries, Developing Countries, Low-Resource Settings, Underdeveloped Countries, Low-Income Countries, Middle-Income Countries, Limited Resource Settings, Africa South of the Sahara, Sub-Saharan Africa, Less Resourced Communities, Afghanistan, Albania, Algeria, American Samoa, Angola, Argentina, Armenia, Azerbaijan, Bangladesh, Belarus, Belize, Benin, Bhutan, Bolivia, Bosnia and Herzegovina, Botswana, Brazil, Bulgaria, Burkina Faso, Burundi, Cabo Verde, Cambodia, Cameroon, Central African Republic, Chad, China, Colombia, Comoros, Democratic Republic of the Congo, DRC,	Antenatal Diagnosis, Prenatal Diagnosis, Antenatal Screening, Prenatal Screening, Antenatal Ultrasound, Prenatal Ultrasound, Antenatal Ultrasonography, Prenatal Ultrasonography

Confidential: For Review Only

Tracheoesophageal Fistula, Intestinal Atresia, Duodenal Obstruction, Duodenal Atresia, Colonic Atresia, Malrotation, Apple Peel Syndrome, Congenital Diaphragmatic Hernias, Gastroschisis, Abdominal Wall Defects, Exomphalos, Omphalocele, Congenital Limb Deformities, Neural Tube Defects, Bronchogenic Cyst, Bronchopulmonary Sequestration, Congenital Cystic Adenomatoid Malformation of Lung, Renal Anomalies, Genito-urinary Anomalies, Maxillofacial Abnormalities, Mouth Abnormalities, Umbilical Hernia, Hirschsprung Disease, Ganglionic Megacolon, Rectosigmoid Aganglionosis, Colonic Aganglionosis, Intestinal Aganglionosis, Volvulus, Intestinal Volvulus	Republic of the Congo, Costa Rica, Cote d'Ivoire, Ivory Coast, Croatia, Cuba, Djibouti, Dominica, Dominican Republic, Ecuador, Egypt, El Salvador, Equatorial Guinea, Eritrea, Ethiopia, Fiji, Gabon, Gambia, Georgia, Ghana, Grenada, Guatemala, Guinea, Guinea-Bissau, Guyana, Haiti, Honduras, India, Indonesia, Islamic Republic of Iran, Iraq, Jamaica, Jordan, Kazakhstan, Kenya, Kiribati, Democratic People's Republic of Korea, Kosovo, Kyrgyz Republic, Lao PDR, Laos, Lebanon, Lesotho, Liberia, Libya, Macedonia Republic, Madagascar, Malawi, Malaysia, Maldives, Mali, Marshall Islands, Mauritania, Mauritius, Mexico, Micronesia, Moldova, Mongolia, Montenegro, Morocco, Mozambique, Myanmar, Namibia, Nauru, Nepal, Nicaragua, Niger, Nigeria, Pakistan, Panama, Papua New Guinea, Paraguay, Peru, Philippines, Romania, Russian Federation, Rwanda, Samoa, Sao Tome and Principe, Senegal, Serbia, Sierra Leone, Solomon Islands, Somalia, Somaliland, South Africa, South Sudan, Sri Lanka, Saint Lucia, Saint Vincent and the Grenadines, Sudan, Suriname, Swaziland, Syrian Arab Republic, Syria, Tajikistan, Tanzania, Thailand, Timor-Leste, East Timor, Togo, Tonga, Tunisia, Turkey, Turkmenistan, Tuvalu, Uganda, Ukraine, Uzbekistan, Vanuatu, Venezuela, Vietnam, West Bank and Gaza, Republic of Yemen, Zambia, Zimbabwe	

Confidential: For Review Only

A further search will be conducted on the WHO website in order to get a more robust understanding of current programs and policies in place for antenatal ultrasound. Researchers will search the following terms in the WHO Reproductive Health Library: ultrasound, ultrasonography, congenital anomalies, congenital abnormalities, congenital anomaly, congenital abnormality, birth defect, antenatal detection, prenatal detection, antenatal diagnosis, and prenatal diagnosis. These terms were chosen based on an initial screening

performed by the researchers which suggested that these terms provided optimum sensitivity for procuring all relevant WHO literature for this review.

Published Literature Search

Using the Ovid program, an electronic database search will be conducted on MEDLINE, EMBASE, Pubmed, and the Cochrane Library using the aforementioned search strategy. These searches will be filtered to only include studies with human subjects that are written in English. An example of the search in MEDLINE can be found in Supplementary File 2.

Inclusion/Exclusion Criteria

Only infants with a structural congenital anomaly as listed in search string 1 will be included. Studies focusing on non-structural congenital anomalies will be excluded. Furthermore, all studies must focus on participants from LMICs; research focusing on high-income countries (HICs) will be excluded. Finally, studies that concentrate on antenatal diagnosis will be included, while studies focusing on postnatal diagnosis will be excluded.

Study Design

No filters were applied to study types; thus, all forms of evidence-based research will be included.

Methodological Quality

To assess the methodological quality of the studies, the researchers will use the Cochrane Risk of Bias for Non-Randomized Studies of Intervention (ROBINS-I) and the revised tool to assess Risk of Bias in randomized trials (RoB 2.0) [13, 14]. These tools are widely used for critically appraising study methodology. For the purposes of this study, these tools will be used as a means of quality assessment, rather than as an inclusion/exclusion determinant. Two independent reviewers will score the articles according to the ROBINS-I and RoB 2.0 criteria and any discrepancies will be assessed by the research team until unanimity is reached.

Study Screening

References from the search results will be added to EndNote X8 and duplicates will be removed. The articles will then be uploaded to Covidence for the screening process. Two reviewers will screen the titles and abstracts in duplicate, removing any articles that do not meet the inclusion criteria. The remaining articles will be assessed by two reviewers independently in full-text and any remaining articles that do not adhere to the study criteria will be removed.

Data Extraction

Confidential: For Review Only

Data extraction will be undertaken by the principal investigator. The following data will be extracted: place of study, study type, publication status, study population, patient cohort, gestational age at time of diagnosis, type of anomaly, type of ultrasound, percent receiving antenatal ultrasound, percent with any anomaly detected, percent with accurate antenatal diagnosis, training of ultrasound technician, referral rate for further antenatal care, referral rate to a tertiary pediatric surgery center, termination rate, mortality rate and complications as per the Clavien-Dindo classification. The following data will be collected regarding antenatal ultrasound policies: date, country/region, governing body, population coverage, intervention(s) and outcomes as detailed above.

Data Synthesis

Descriptive statistics and narrative synthesis will be utilized. Data will be presented according to the following categories: effectiveness of antenatal ultrasound, percent of women receiving antenatal ultrasound, training of personnel performing ultrasound examination, and morbidity and mortality outcomes.

Based on the authors' experience, it is unlikely that a meta-analysis will be feasible due to limited availability of data. However, if there is appropriate data, a meta-analysis will be undertaken in duplicate by two independent authors and discrepancies resolved amongst the wider authorship. Appropriate data will be defined as: two or more sufficiently homogeneous studies comparing morbidity and/ or mortality between one group who has received antenatal ultrasound and another who has not. In addition, a meta-analysis may be undertaken if two or more studies have compared an intervention to improve antenatal ultrasound coverage in a population or detection rates. Meta-analysis will be undertaken in Stata and results presented using a forest plot. If there are over 10 studies in the meta-analysis a funnel plot will be undertaken to assess publication bias and a Galbraith plot to investigate heterogeneity in effect sizes. The quality of evidence will be assessed following GRADE guidelines.

DISCUSSION

The discrepancy in mortality due to congenital anomalies between HICs and LMICs is quite substantial. For instance, the survival rate for infants with gastroschisis in HICs is above 95%, while in many LMICs, there are few survivors of the condition [10]. This study aims to investigate the use of antenatal ultrasound to diagnose congenital anomalies in LMICs. Further, this study will examine the current policies and programs in place for antenatal ultrasound. Gaining a better understanding of the current policies and practices which increase the antenatal diagnosis of structural congenital anomalies may help to determine the most effective standards of practice. Increasing the early diagnosis of structural congenital anomalies may help to reduce the morbidity and mortality rates of congenital anomalies in LMICs. Many complications that arise from certain structural congenital anomalies could be avoided if the condition is detected antenatally and steps are taken to ensure safe delivery, such as planning

Confidential: For Review Only

the birth at a tertiary healthcare facility. This study may provide crucial information regarding the effects of antenatal ultrasound on morbidity and mortality rates from congenital anomalies in LMICs. This in turn can inform future studies aimed at improving availability and quality of antenatal ultrasound in LMICs to ultimately improve the health outcomes of infants born with these conditions.

ETHICS AND DISSEMINATION

The researchers of this study will be conducting a secondary analysis. No new data will be collected and there will be no direct interaction with participants. Therefore, it is not necessary to acquire ethical approval prior to conducting this review. Following data collection and analysis, the results will be submitted to a peer-reviewed scientific journal for publication. The results will also be shared in conferences pertaining to infant health, global health and global surgery.

Confidential: For Review Only

References

1. Apfeld, J.C., et al., *Infant, maternal, and geographic factors influencing gastroschisis related mortality in Zimbabwe.* Surgery, 2015. **158**(6): p. 1475-80.
2. Sitkin, N.A., et al., *Congenital anomalies in low- and middle-income countries: the unborn child of global surgery.* World J Surg, 2015. **39**(1): p. 36-40.
3. Collaborators, G.B.D.C.M., *Global, regional, national, and selected subnational levels of stillbirths, neonatal, infant, and under-5 mortality, 1980-2015: a systematic analysis for the Global Burden of Disease Study 2015.* Lancet, 2016. **388**(10053): p. 1725-1774.
4. Wu, V.K., D. Poenaru, and M.J. Poley, *Burden of surgical congenital anomalies in Kenya: a population-based study.* J Trop Pediatr, 2013. **59**(3): p. 195-202.
5. Ford, K., et al., *Gastroschisis: Bellwether for neonatal surgery capacity in low resource settings?* J Pediatr Surg, 2016. **51**(8): p. 1262-7.
6. Mashuda, F., et al., *Pattern and factors associated with congenital anomalies among young infants admitted at Bugando medical centre, Mwanza, Tanzania.* BMC Res Notes, 2014. **7**: p. 195.
7. *Congenital Anomalies*. 2016; Available from: http://www.who.int/mediacentre/factsheets/fs370/en/.
8. *What are the types of birth defects?* 2017; Available from: https://www.nichd.nih.gov/health/topics/birthdefects/conditioninfo/types.

Confidential: For Review Only

9. Payne, N.R., et al., *Predicting the Outcome of Newborns with Gastroschisis.* Journal of pediatric surgery, 2009. **44**(5): p. 918-923.

10. Badrinath, R., et al., *Outcomes and unmet need for neonatal surgery in a resource-limited environment: estimates of global health disparities from Kampala, Uganda.* J Pediatr Surg, 2014. **49**(12): p. 1825-30.

11. Moher, D., et al., *Preferred reporting items for systematic review and meta-analysis protocols (PRISMA-P) 2015 statement.* Syst Rev, 2015. **4**: p. 1.

12. Shamseer, L., et al., *Preferred reporting items for systematic review and meta-analysis protocols (PRISMA-P) 2015: elaboration and explanation.* BMJ, 2015. **350**: p. g7647.

13. Higgins, J., et al., *Appraising the risk of bias in randomized trials using the Cochrane Risk of Bias Tool.* Cochrane Database of Systematic Reviews, 2016. **10**(Suppl 1).

14. Sterne, J.A., et al., *ROBINS-I: a tool for assessing risk of bias in non-randomised studies of interventions.* BMJ, 2016. **355**: p. i4919.

Confidential: For Review Only

Acknowledgements

We would like to thank the library services at King's College London for help with the systematic review process.

Contributors

NW conceived the idea for this study. All authors devised the study design. SG drafted the protocol with significant contributions from NW and AK. SG and SSB will perform the literature review and SG will draft the results.

Funding

NW receives funding from the Wellcome Trust to undertake a Clinical PhD in Global Health at King's Centre for Global Health and Health Partnerships, King's College London.

Competing Interests

The authors have no competing interests to declare.

Confidential: For Review Only

Supplementary File 1

Investigating the Use of Ultrasonography for the Antenatal Diagnosis of Structural Congenital Anomalies in Low- and Middle-Income Countries: A Systematic Review Protocol

PRISMA-P (Preferred Reporting Items for Systematic review and Meta-Analysis Protocols) 2015 Checklist

Section and topic	Item No	Checklist item	Section Where Addressed in Protocol
**Administrative information**			
Title:			
Identification	1a	Identify the report as a protocol of a systematic review	Title Page
Update	1b	If the protocol is for an update of a previous systematic review, identify as such	N/A
Registration	2	If registered, provide the name of the registry (such as PROSPERO) and registration number	Title Page
Authors:			
Contact	3a	Provide name, institutional affiliation, e-mail address of all protocol authors; provide physical mailing address of corresponding author	Title Page
Contributions	3b	Describe contributions of protocol authors and identify the guarantor of the review	Contributors
Amendments	4	If the protocol represents an amendment of a previously completed or published protocol, identify as such and list changes; otherwise, state plan for documenting important protocol amendments	Methods and Analysis
Support:			
Sources	5a	Indicate sources of financial or other support for the review	Funding
Sponsor	5b	Provide name for the review funder and/or sponsor	Funding
Role of sponsor or funder	5c	Describe roles of funder(s), sponsor(s), and/or institution(s), if any, in developing the protocol	Funding
**Introduction**			
Rationale	6	Describe the rationale for the review in the context of what is already known	Introduction
Objectives	7	Provide an explicit statement of the question(s) the review will address with reference to participants, interventions, comparators, and outcomes (PICO)	Aim; Objectives
**Methods**			
Eligibility criteria	8	Specify the study characteristics (such as PICO, study design, setting, time frame) and report characteristics (such as years considered, language, publication status) to be used as criteria for eligibility for the review	Search Strategy; Inclusion/Exclusion Criteria
Information sources	9	Describe all intended information sources (such as electronic databases, contact with study authors, trial registers or other grey literature sources) with planned dates of coverage	Published Literature Search

Confidential: For Review Only

Section and topic	Item No	Checklist item	Section Where Addressed in Protocol
Search strategy	10	Present draft of search strategy to be used for at least one electronic database, including planned limits, such that it could be repeated	Search Strategy; Table 1; Supplementary Document 2
Study records:			
Data management	11a	Describe the mechanism(s) that will be used to manage records and data throughout the review	Study Screening; Data Extraction
Selection process	11b	State the process that will be used for selecting studies (such as two independent reviewers) through each phase of the review (that is, screening, eligibility and inclusion in meta-analysis)	Study Screening
Data collection process	11c	Describe planned method of extracting data from reports (such as piloting forms, done independently, in duplicate), any processes for obtaining and confirming data from investigators	Data Extraction
Data items	12	List and define all variables for which data will be sought (such as PICO items, funding sources), any pre-planned data assumptions and simplifications	Data Extraction
Outcomes and prioritization	13	List and define all outcomes for which data will be sought, including prioritization of main and additional outcomes, with rationale	Data Extraction
Risk of bias in individual studies	14	Describe anticipated methods for assessing risk of bias of individual studies, including whether this will be done at the outcome or study level, or both; state how this information will be used in data synthesis	Methodological Quality
Data synthesis	15a	Describe criteria under which study data will be quantitatively synthesized	Data Synthesis
	15b	If data are appropriate for quantitative synthesis, describe planned summary measures, methods of handling data and methods of combining data from studies, including any planned exploration of consistency (such as I^2, Kendall's τ)	Data Synthesis
	15c	Describe any proposed additional analyses (such as sensitivity or subgroup analyses, meta-regression)	N/A
	15d	If quantitative synthesis is not appropriate, describe the type of summary planned	Data Synthesis
Meta-bias(es)	16	Specify any planned assessment of meta-bias(es) (such as publication bias across studies, selective reporting within studies)	Data Synthesis
Confidence in cumulative evidence	17	Describe how the strength of the body of evidence will be assessed (such as GRADE)	Data Synthesis

From: Shamseer L, Moher D, Clarke M, Ghersi D, Liberati A, Petticrew M, Shekelle P, Stewart L, PRISMA-P Group. Preferred reporting items for systematic review and meta-analysis protocols (PRISMA-P) 2015: elaboration and explanation. BMJ. 2015 Jan 2;349(jan02 1):g7647.

Confidential: For Review Only

Supplementary File 2

Investigating the Use of Ultrasonography for the Antenatal Diagnosis of Structural Congenital Anomalies in Low- and Middle-Income Countries: A Systematic Review Protocol

Database: **Ovid MEDLINE(R) Epub Ahead of Print, In-Process & Other Non-Indexed Citations, Ovid MEDLINE(R) Daily, Ovid MEDLINE and Versions(R)** <1946 to June 27, 2018>

Confidential: For Review Only

Search Strategy:

No.	Search Terms	Results
1	exp Congenital Abnormalities/ or congenital anomal*.mp.	559922
2	exp Congenital Abnormalities/ or Congenital Abnormal*.mp.	555311
3	Congenital Malformation*.mp.	13826
4	Fetal Malformation*.mp.	1917
5	Birth Defect*.mp.	9122
6	Anencephaly.mp. or exp ANENCEPHALY/	3875
7	Conjoined Twins.mp. or exp Twins, Conjoined/	2022
8	exp Heart Defects, Congenital/ or Congenital Heart Defect*.mp.	142854
9	Anorectal Malformation*.mp.	1896
10	Anal Stenosis.mp.	389
11	Anal Atresia.mp. or exp Anus, Imperforate/	2815
12	Imperforate Anus.mp. or exp Anus, Imperforate/	2992
13	Biliary atresia.mp. or exp Biliary Atresia/	4482
14	exp Choledochal Cyst/ or Choledochal Cyst*.mp.	2825
15	exp Diaphragmatic Eventration/ or Diaphragmatic Eventration*.mp.	943
16	Esophageal Atresia.mp. or exp Esophageal Atresia/	4173
17	exp Tracheoesophageal Fistula/ or Tracheo-?esophageal fistula*.mp.	3676
18	Intestinal Atresia.mp. or exp Intestinal Atresia/	2301
19	Duodenal atresia.mp.	839
20	Colonic Atresia.mp.	152
21	Malrotation.mp.	2393
22	Apple peel syndrome.mp.	16
23	exp Hernias, Diaphragmatic, Congenital/ or congenital diaphragmatic hernia*.mp.	5530
24	Gastroschisis.mp. or exp GASTROSCHISIS/	2313
25	Abdominal Wall Defect*.mp.	2009
26	Exomphalos.mp. or exp Hernia, Umbilical/	3740
27	Omphalocele.mp. or exp Hernia, Umbilical/	4242
28	Congenital Limb Deformities.mp. or exp Limb Deformities, Congenital/	22483
29	exp Neural Tube Defects/ or Neural Tube Defect*.mp.	29051
30	exp Bronchogenic Cyst/ or Bronchogenic Cyst*.mp.	1810
31	exp Bronchopulmonary Sequestration/ or Bronchopulmonary sequestration*.mp.	2022
32	exp "Cystic Adenomatoid Malformation of Lung, Congenital"/ or Congenital Cystic adenomatoid malformation of lung*.mp.	874
33	Renal Anomal*.mp.	1185
34	exp Urogenital Abnormalities/ or Genito-urinary Anomalies.mp.	57735
35	exp Maxillofacial Abnormalities/ or Maxillofacial Abnormalit*.mp.	28265
36	exp Mouth Abnormalities/ or Mouth Abnormalit*.mp.	24459
37	Umbilical Hernia.mp. or exp Hernia, Umbilical/	4200
38	Hirschsprung disease.mp. or exp Hirschsprung Disease/	4567
39	exp Megacolon/ or Ganglionic Megacolon.mp.	7053
40	Rectosigmoid aganglionosis.mp.	15
41	Colonic Aganglionosis.mp.	311
42	Intestinal Aganglionosis.mp.	190
43	Volvulus.mp. or exp Intestinal Volvulus/	8927
44	LMICs.mp.	2001
45	(low- and middle-income countr*).mp. [mp=title, abstract, original title, name of substance word, subject heading word, floating sub-heading word, keyword heading word, protocol supplementary concept word, rare disease supplementary concept word, unique identifier, synonyms]	10910
46	Low-resource setting*.mp.	2719

47	Underdeveloped Countries.mp.	817
48	low-income countries.mp.	4707
49	exp Developing Countries/ or Developing countr*.mp.	115302
50	middle-income countries.mp.	11136
51	limited resource setting*.mp.	253
52	Africa South of the Sahara.mp. or exp "Africa South of the Sahara"/	184102
53	Sub-Saharan Africa.mp. or exp "Africa South of the Sahara"/	190396
54	Less Resourced communities.mp.	1
55	Afghanistan.mp. or exp AFGHANISTAN/	6065
56	Albania.mp. or exp ALBANIA/	1182
57	Algeria.mp. or exp ALGERIA/	3621
58	American Samoa.mp.	354
59	Angola.mp. or exp ANGOLA/	1403
60	Argentina.mp. or exp ARGENTINA/	19610
61	Armenia.mp. or exp ARMENIA/	1676
62	Azerbaijan.mp. or exp AZERBAIJAN/	1765
63	Bangladesh.mp. or exp BANGLADESH/	12907
64	Belarus.mp. or exp "Republic of Belarus"/	2553
65	Belize.mp. or exp BELIZE/	868
66	Benin.mp. or exp BENIN/	3441
67	exp BHUTAN/ or Bhutan.mp.	628
68	Bolivia.mp. or exp BOLIVIA/	3594
69	(Bosnia and Herzegovina).mp. [mp=title, abstract, original title, name of substance word, subject heading word, floating sub-heading word, keyword heading word, protocol supplementary concept word, rare disease supplementary concept word, unique identifier, synonyms]	2477
70	Botswana.mp. or exp BOTSWANA/	2247
71	Brazil.mp. or exp BRAZIL/	101919
72	Bulgaria.mp. or exp BULGARIA/	7554
73	Burkina Faso.mp. or exp Burkina Faso/	3904
74	Burundi.mp. or exp BURUNDI/	871
75	Cabo Verde.mp. or exp Cabo Verde/	211
76	Cambodia.mp. or exp CAMBODIA/	4102
77	Cameroon.mp. or exp CAMEROON/	6804
78	Central African Republic.mp. or exp Central African Republic/	1096
79	Chad.mp. or exp CHAD/	1144
80	China.mp. or exp CHINA/	205799
81	Colombia.mp. or exp COLOMBIA/	12533
82	Comoros.mp. or exp COMOROS/	427
83	Democratic Republic of the Congo.mp. or exp "Democratic Republic of the Congo"/	4506
84	exp "Democratic Republic of the Congo"/ or DRC.mp.	4827
85	Republic of the Congo.mp. or exp Congo/	6176
86	Costa Rica.mp. or exp Costa Rica/	5088
87	Cote d'Ivoire.mp. or exp Cote d'Ivoire/	3527
88	Ivory Coast.mp. or exp Cote d'Ivoire/	3432
89	Croatia.mp. or exp CROATIA/	8261
90	Cuba.mp. or exp CUBA/	6343
91	Djibouti.mp. or exp DJIBOUTI/	372
92	Dominica.mp. or exp DOMINICA/	430
93	Dominican Republic.mp. or exp Dominican Republic/	2128
94	Ecuador.mp. or exp ECUADOR/	4722
95	exp EGYPT/ or Egypt.mp.	18702
96	El Salvador.mp. or exp El Salvador/	1319
97	Equatorial Guinea.mp. or exp Equatorial Guinea/	419
98	Eritrea.mp. or exp ERITREA/	518
99	Ethiopia.mp. or exp ETHIOPIA/	13354
100	Fiji.mp. or exp FIJI/	1620
101	Gabon.mp. or exp GABON/	1925
102	exp GAMBIA/ or Gambia.mp.	2958
103	exp "GEORGIA (REPUBLIC)"/ or exp GEORGIA/ or Georgia.mp.	15842
104	Ghana.mp. or exp GHANA/	9432
105	Grenada.mp. or exp GRENADA/	308
106	Guatemala.mp. or exp GUATEMALA/	3907
107	Guinea.mp. or exp GUINEA-BISSAU/ or exp EQUATORIAL GUINEA/ or exp GUINEA/ or exp PAPUA NEW GUINEA/ or exp NEW GUINEA/	160074
108	Guyana.mp. or exp GUYANA/	1136
109	Haiti.mp. or exp HAITI/	3707
110	Honduras.mp. or exp HONDURAS/	1736

Confidential: For Review Only

111	India.mp. or exp INDIA/	129215
112	Indonesia.mp. or exp INDONESIA/	13107
113	exp Iran/ or Islamic Republic of Iran.mp.	21280
114	exp IRAQ/ or Iraq.mp.	9035
115	Jamaica.mp. or exp JAMAICA/	4427
116	Jordan.mp. or exp JORDAN/	6040
117	Kazakhstan.mp. or exp KAZAKHSTAN/	3153
118	Kenya.mp. or exp KENYA/	18798
119	Kiribati.mp. or exp Micronesia/	1934
120	Democratic People's Republic of Korea.mp. or exp "Democratic People's Republic of Korea"/	246
121	Kosovo.mp. or exp KOSOVO/	840
122	Kyrgyz Republic.mp. or exp Kyrgyzstan/	1225
123	exp Laos/ or Lao PDR.mp.	1876
124	Laos.mp. or exp LAOS/	2431
125	Lebanon.mp. or exp LEBANON/	5011
126	Lesotho.mp. or exp LESOTHO/	645
127	Liberia.mp. or exp LIBERIA/	1667
128	Libya.mp. or exp LIBYA/	1554
129	Macedonia Republic.mp. or exp "Macedonia (Republic)"/	475
130	Madagascar.mp. or exp MADAGASCAR/	4734
131	Malawi.mp. or exp MALAWI/	6317
132	Malaysia.mp. or exp MALAYSIA/	18390
133	Maldives.mp. or exp Indian Ocean Islands/	10595
134	Mali.mp. or exp MALI/	3511
135	Marshall Islands.mp. or exp Micronesia/	1959
136	Mauritania.mp. or exp MAURITANIA/	626
137	Mauritius.mp. or exp MAURITIUS/	953
138	exp MEXICO/ or Mexico.mp.	53282
139	Micronesia.mp. or exp MICRONESIA/	2243
140	Moldova.mp. or exp MOLDOVA/	920
141	Mongolia.mp. or exp MONGOLIA/	3809
142	Montenegro.mp. or exp MONTENEGRO/	749
143	Morocco.mp. or exp MOROCCO/	6855
144	Mozambique.mp. or exp MOZAMBIQUE/	3236
145	Myanmar.mp. or exp MYANMAR/	3198
146	Namibia.mp. or exp NAMIBIA	1502
147	Nauru.mp. or exp Micronesia/	1921
148	Nepal.mp. or exp NEPAL/	9337
149	Nicaragua.mp. or exp NICARAGUA/	1949
150	exp NIGER/ or Niger.mp.	12299
151	Nigeria.mp. or exp NIGERIA/	32627
152	Pakistan.mp. or exp PAKISTAN/	20153
153	exp PANAMA/ or Panama.mp.	4265
154	Papua New Guinea.mp. or exp Papua New Guinea/	5003
155	Paraguay.mp. or exp PARAGUAY/	1433
156	Peru.mp. or exp PERU/	11249
157	Philippines.mp. or exp PHILIPPINES/	10698
158	Romania.mp. or exp ROMANIA/	11521
159	exp Russia/ or Russian Federation.mp.	51376
160	Rwanda.mp. or exp RWANDA/	2883
161	exp AMERICAN SAMOA/ or Samoa.mp. or exp SAMOA/ or exp "INDEPENDENT STATE OF SAMOA"/	1024
162	(Sao Tome and Principe).mp. [mp=title, abstract, original title, name of substance word, subject heading word, floating sub-heading word, keyword heading word, protocol supplementary concept word, rare disease supplementary concept word, unique identifier, synonyms]	135
163	Senegal.mp. or exp SENEGAL/	7067
164	Serbia.mp. or exp SERBIA/	4574
165	Sierra Leone.mp. or exp Sierra Leone/	2103
166	Solomon Islands.mp. or exp Melanesia/	6193
167	Somalia.mp. or exp SOMALIA/	1968
168	Somaliland.mp.	136
169	South Africa.mp. or exp South Africa/	47075
170	South Sudan.mp. or exp South Sudan/	397
171	Sri Lanka.mp. or exp Sri Lanka/	7342
172	Saint Lucia.mp. or exp Saint Lucia/	114
173	(Saint Vincent and the Grenadines).mp. [mp=title, abstract, original title, name of substance word, subject heading word, floating sub-heading word, keyword heading word, protocol supplementary concept word, rare disease supplementary concept word, unique identifier, synonyms]	57

For confidential: For Review Only

174	exp SUDAN/ or Sudan.mp.	8429
175	Suriname.mp. or exp SURINAME/	1098
176	Swaziland.mp. or exp SWAZILAND/	818
177	Syrian Arab Republic.mp.	73
178	Syria.mp. or exp SYRIA/	2407
179	Tajikistan.mp. or exp TAJIKISTAN/	910
180	Tanzania.mp. or exp TANZANIA/	12921
181	Thailand.mp. or exp THAILAND/	32186
182	exp Timor-Leste/ or Timor-Leste.mp.	311
183	East Timor.mp.	187
184	Togo.mp. or exp TOGO/	1491
185	Tonga.mp. or exp TONGA/	463
186	Tunisia.mp. or exp TUNISIA/	9057
187	exp TURKEY/ or Turkey.mp.	46898
188	Turkmenistan.mp. or exp TURKMENISTAN/	717
189	Tuvalu.mp. or exp Micronesia/	1884
190	Uganda.mp. or exp UGANDA/	13956
191	Ukraine.mp. or exp UKRAINE/	16702
192	Uzbekistan.mp. or exp UZBEKISTAN/	2161
193	Vanuatu.mp. or exp VANUATU/	629
194	Venezuela.mp. or exp VENEZUELA/	6620
195	exp VIETNAM/ or Vietnam.mp.	15919
196	(West Bank and Gaza).mp. [mp=title, abstract, original title, name of substance word, subject heading word, floating sub-heading word, keyword heading word, protocol supplementary concept word, rare disease supplementary concept word, unique identifier, synonyms]	194
197	Republic of Yemen.mp. or exp Yemen/	1264
198	Zambia.mp. or exp ZAMBIA/	5404
199	Zimbabwe.mp. or exp ZIMBABWE/	6879
200	Antenatal Diagnosis.mp.	2383
201	Prenatal Diagnosis.mp. or exp Prenatal Diagnosis/	74636
202	Antenatal Screening.mp.	1193
203	Prenatal Screening.mp.	2579
204	Antenatal Ultrasound.mp.	702
205	Prenatal Ultrasound.mp.	2203
206	Antenatal Ultrasonography.mp.	200
207	Prenatal Ultrasonography.mp. or exp Ultrasonography, Prenatal/	30427
208	1 or 2 or 3 or 4 or 5 or 6 or 7 or 8 or 9 or 10 or 11 or 12 or 13 or 14 or 15 or 16 or 17 or 18 or 19 or 20 or 21 or 22 or 23 or 24 or 25 or 26 or 27 or 28 or 29 or 30 or 31 or 32 or 33 or 34 or 35 or 36 or 37 or 38 or 39 or 40 or 41 or 42 or 43	607001
209	44 or 45 or 46 or 47 or 48 or 49 or 50 or 51 or 52 or 53 or 54 or 55 or 56 or 57 or 58 or 59 or 60 or 61 or 62 or 63 or 64 or 65 or 66 or 67 or 68 or 69 or 70 or 71 or 72 or 73 or 74 or 75 or 76 or 77 or 78 or 79 or 80 or 81 or 82 or 83 or 84 or 85 or 86 or 87 or 88 or 89 or 90 or 91 or 92 or 93 or 94 or 95 or 96 or 97 or 98 or 99 or 100 or 101 or 102 or 103 or 104 or 105 or 106 or 107 or 108 or 109 or 110 or 111 or 112 or 113 or 114 or 115 or 116 or 117 or 118 or 119 or 120 or 121 or 122 or 123 or 124 or 125 or 126 or 127 or 128 or 129 or 130 or 131 or 132 or 133 or 134 or 135 or 136 or 137 or 138 or 139 or 140 or 141 or 142 or 143 or 144 or 145 or 146 or 147 or 148 or 149 or 150 or 151 or 152 or 153 or 154 or 155 or 156 or 157 or 158 or 159 or 160 or 161 or 162 or 163 or 164 or 165 or 166 or 167 or 168 or 169 or 170 or 171 or 172 or 173 or 174 or 175 or 176 or 177 or 178 or 179 or 180 or 181 or 182 or 183 or 184 or 185 or 186 or 187 or 188 or 189 or 190 or 191 or 192 or 193 or 194 or 195 or 196 or 197 or 198 or 199 (1385344)	1385344
210	200 or 201 or 202 or 203 or 204 or 205 or 206 or 207	77525
211	208 and 209 and 210	896

Confidential: For Review Only

BMJ Paediatrics Open

Investigating the Use of Ultrasonography for the Antenatal Diagnosis of Structural Congenital Anomalies in Low- and Middle-Income Countries: A Systematic Review Protocol

Journal:	*BMJ Paediatrics Open*
Manuscript ID	bmjpo-2019-000538.R1
Article Type:	Protocol
Date Submitted by the Author:	14-Aug-2019
Complete List of Authors:	Goley, Stephanie; King's College London, Department of Global Health & Social Medicine<br>Sakula-Barry, Sidonie; World Cancer Research Fund<br>Kelly, Ann; King's College London, Department of Global Health & Social Medicine<br>Wright, Naomi; King's College London, King's Centre for Global Health and Health Partnerships
Keywords:	Congenital Abnorm, Paediatric Surgery, Neonatology, Imaging

SCHOLARONE™
Manuscripts

Investigating the Use of Ultrasonography for the Antenatal Diagnosis of Structural Congenital Anomalies in Low- and Middle-Income Countries: A Systematic Review Protocol

Stephanie Goley[1], Sidonie Sakula-Barry[2], Ann Kelly[1], Naomi Wright[3]

[1] Department of Global Health & Social Medicine, King's College London, London, UK
[2] World Cancer Research Fund UK, London, UK
[3] King's Centre for Global Health and Health Partnerships, School of Population Health and Environmental Sciences, King's College London, London, UK

Authors' Email Addresses:
info@stephgoley.com
sidonie.sakula-barry1@alumni.lshtm.ac.uk
ann.kelly@kcl.ac.uk
naomiwright@doctors.org.uk

Corresponding Author:
Stephanie Goley, BA MSc
Department of Global Health & Social Medicine, King's College London, London, UK
Correspondence to: Centre for Global Health and Health Partnerships, Suite 2.13 Weston Education Centre, Cutcombe Road, London, SE5 9RJ
Telephone: +1 (815) 830-5360

PROSPERO Registration: CRD42019105620
Registered on February 14, 2019
Prepared according to Prisma-P Guidelines

Confidential: For Review Only

**What is known?**
• 97% of deaths from congenital anomalies occur in developing countries. Many of these deaths could be prevented with early diagnosis and intervention.<br>• Ultrasound machines are widely accessible and commonplace in HICs, but a number of factors limit the accessibility and effectiveness of ultrasound in LMICs.
**What will this study add?**
• To systematically investigate, for the first time, the availability and effectiveness of antenatal ultrasound in LMICs in order to elucidate disparities in congenital anomaly detection.<br>• To evaluate the effects of antenatal ultrasound on the morbidity and mortality rates of neonates with a structural congenital anomaly in LMICs.

ABSTRACT

Introduction: Congenital anomalies are the 5th leading cause of under-5 mortality globally. The greatest burden is faced by those in developing countries, where over 95% of deaths occur. Many of these deaths may have been preventable through antenatal diagnosis and early intervention. This study aims to conduct a systematic review that investigates the use of antenatal ultrasound to diagnose congenital anomalies and improve the health outcomes of infants in low- and middle-income countries (LMICs).

Methods and Analysis: A systematic literature review will be conducted using three search strings: 1) structural congenital anomalies; 2) LMICs; and 3) antenatal diagnosis. Four electronic databases will be searched: MEDLINE, EMBASE, PubMed, and the Cochrane Library. Title, abstract and full text screening will be undertaken in duplicate by two reviewers independently using Covidence. Consensus will be sought from the wider authorship for discrepancies. Data extraction will be undertaken by the principal investigator. The primary analysis will focus on the availability and effectiveness of antenatal ultrasound for structural congenital anomalies. Secondary outcomes will include neonatal morbidity and mortality, termination rates, and referral rates for further antenatal care. Descriptive statistics and a narrative synthesis will be included in the final report. Methodological quality of the included studies will be evaluated using the Cochrane-approved ROBINS-I and RoB 2.0 tools.

Ethics and Dissemination: Ethical approval is not required for conducting the systematic review as there will be no direct collection of data from individuals. The results will be submitted for publication in a scientific journal and presented internationally.

Conclusion: This is the first study, to our knowledge, to systematically review current literature on the use of antenatal ultrasound for the detection of congenital anomalies in LMICs. This is vital to define current practice, highlight global disparities and evaluate effects on health outcomes for infants in low-resource settings.

Confidential: For Review Only

INTRODUCTION

Congenital anomalies represent 9% of the global burden of surgical disease and are one of the leading causes of infant morbidity and mortality globally [1-4]. The burden of congenital anomalies is much greater in low- and middle-income countries (LMICs), comprising over 95% of deaths from these conditions [5, 6]. According to a report by the WHO, every year approximately 303,000 infants die within 4 weeks of birth due to congenital anomalies [7]. However, this is likely an underestimation, as statistics are undoubtedly skewed from underreporting, a lack of congenital anomaly registries, and unreliable medical records in many LMICs.

Structural congenital anomalies are physical deformities that occur during intrauterine development [7]. These anomalies include, but are not limited to, gastro-intestinal malformations, cleft lip and palate, heart defects, musculoskeletal anomalies, and neural tube defects [8]. If detected early and provided appropriate surgical intervention following birth, many structural congenital anomalies can be corrected avoiding preventable death or disability [7].

Advancements in ultrasound technology have allowed for early detection of structural congenital anomalies, which allows mothers and physicians to plan for an appropriate place of delivery and surgical intervention after birth [9]. In many cases, this can result in a significantly reduced risk of morbidity and mortality [10]. While ultrasound machines are widely accessible and commonplace in high-income countries, a number of factors limit the access and effectiveness of antenatal ultrasound in LMICs.

This study aims to systematically investigate, for the first time, the availability and effectiveness of antenatal ultrasound in the diagnosis of structural congenital anomalies in LMICs. Furthermore it aims to evaluate the effects of antenatal ultrasound on mortality and morbidity for neonates with a structural congenital anomaly in LMICs. Such information is vital to help clarify the existing disparities in antenatal ultrasound provision and the potential benefits for improved health outcomes through the delivery of this service in low-resource settings.

METHODS

PRISMA-P guidelines will be followed in conducting this systematic review [11, 12] (Supplementary File 1). If there are amendments to the protocol, they will be reported in the publication of the results.

Aim

To conduct a systematic review that investigates the use of antenatal ultrasound to diagnose congenital anomalies and improve the health outcomes of infants in low- and middle-income countries.

Confidential: For Review Only

Objectives

1. To systematically identify and describe studies that focus on the antenatal diagnosis of structural congenital anomalies in LMICs.
2. To evaluate the utilization and effectiveness of ultrasound machines in the antenatal diagnosis of structural congenital anomalies and to report the current practices and policies regarding congenital anomaly detection in LMICs.
3. To evaluate the effects of antenatal ultrasound on the morbidity and mortality rates of neonates with a structural congenital anomaly in LMICs.
4. To critique the methodological quality of the included articles.

Patient and Public Involvement

Given that this is a systematic literature review, there will be no patient or public involvement for the data collection and review of the literature. However, public involvement will be important for prioritizing antenatal ultrasound on the political agenda. It will also be crucial for improving current antenatal healthcare programs. Following conclusion of this study, international parent/patient support groups and charities involving structural congenital anomalies will be approached to assist with the dissemination of the findings via their websites, social media, in person meetings and other appropriate routes. A plain English summary of the findings will be provided for this purpose.

Search Strategy

A search will be conducted using three search strings (Table 1). Search string 1 will encompass structural congenital anomalies. Search string 2 will focus on the setting - LMICs. Finally, search string 3 will look at the antenatal diagnosis of structural congenital anomalies, particularly focusing on the use of ultrasound machines for detection. Borlean operators 'and' and 'or' will be utilized within the search to combine the search terms.

Table 1: Search Strings

Search String 1	Search String 2	Search String 3
Congenital Anomalies, Congenital Abnormalities, Congenital Malformation, Fetal Malformation, Birth Defects, Anencephaly, Conjoined Twins, Congenital Heart Defects, Anorectal Malformations, Anal	LMICs, Low- and Middle-Income Countries, Developing Countries, Low-Resource Settings, Underdeveloped Countries, Low-Income Countries, Middle-Income Countries, Limited Resource Settings, Africa South of the Sahara, Sub-Saharan Africa, Less Resourced Communities, Afghanistan, Albania, Algeria, American Samoa, Angola, Argentina, Armenia, Azerbaijan, Bangladesh, Belarus, Belize, Benin,	Antenatal Diagnosis, Prenatal Diagnosis, Antenatal Screening, Prenatal Screening, Antenatal Ultrasound, Prenatal Ultrasound, Antenatal Ultrasonography, Prenatal Ultrasonography

Confidential: For Review Only

Stenosis, Anal Atresia, Imperforate Anus, Biliary Atresia, Choledochal Cyst, Diaphragmatic Eventration, Esophageal Atresia, Tracheoesophageal Fistula, Intestinal Atresia, Duodenal Obstruction, Duodenal Atresia, Colonic Atresia, Malrotation, Apple Peel Syndrome, Congenital Diaphragmatic Hernias, Gastroschisis, Abdominal Wall Defects, Exomphalos, Omphalocele, Congenital Limb Deformities, Neural Tube Defects, Bronchogenic Cyst, Bronchopulmonary Sequestration, Congenital Cystic Adenomatoid Malformation of Lung, Renal Anomalies, Genito-urinary Anomalies, Maxillofacial Abnormalities, Mouth Abnormalities, Umbilical Hernia, Hirschsprung Disease, Ganglionic Megacolon, Rectosigmoid Aganglionosis, Colonic Aganglionosis, Intestinal Aganglionosis, Volvulus, Intestinal Volvulus	Bhutan, Bolivia, Bosnia and Herzegovina, Botswana, Brazil, Bulgaria, Burkina Faso, Burundi, Cabo Verde, Cambodia, Cameroon, Central African Republic, Chad, China, Colombia, Comoros, Democratic Republic of the Congo, DRC, Republic of the Congo, Costa Rica, Cote d'Ivoire, Ivory Coast, Croatia, Cuba, Djibouti, Dominica, Dominican Republic, Ecuador, Egypt, El Salvador, Equatorial Guinea, Eritrea, Ethiopia, Fiji, Gabon, Gambia, Georgia, Ghana, Grenada, Guatemala, Guinea, Guinea-Bissau, Guyana, Haiti, Honduras, India, Indonesia, Islamic Republic of Iran, Iraq, Jamaica, Jordan, Kazakhstan, Kenya, Kiribati, Democratic People's Republic of Korea, Kosovo, Kyrgyz Republic, Lao PDR, Laos, Lebanon, Lesotho, Liberia, Libya, Macedonia Republic, Madagascar, Malawi, Malaysia, Maldives, Mali, Marshall Islands, Mauritania, Mauritius, Mexico, Micronesia, Moldova, Mongolia, Montenegro, Morocco, Mozambique, Myanmar, Namibia, Nauru, Nepal, Nicaragua, Niger, Nigeria, Pakistan, Panama, Papua New Guinea, Paraguay, Peru, Philippines, Romania, Russian Federation, Rwanda, Samoa, Sao Tome and Principe, Senegal, Serbia, Sierra Leone, Solomon Islands, Somalia, Somaliland, South Africa, South Sudan, Sri Lanka, Saint Lucia, Saint Vincent and the Grenadines, Sudan, Suriname, Swaziland, Syrian Arab Republic, Syria, Tajikistan, Tanzania, Thailand, Timor-Leste, East Timor, Togo, Tonga, Tunisia, Turkey, Turkmenistan, Tuvalu, Uganda, Ukraine, Uzbekistan, Vanuatu, Venezuela, Vietnam, West Bank and Gaza, Republic of Yemen, Zambia, Zimbabwe	

Confidential: For Review Only

A further search will be conducted on the WHO website in order to get a more robust understanding of current programs and policies in place for antenatal ultrasound. Researchers will initially search the following terms in the WHO Reproductive Health Library: ultrasound, ultrasonography, congenital anomalies, congenital abnormalities, congenital anomaly, congenital abnormality, birth defect, antenatal detection, prenatal detection, antenatal diagnosis, and prenatal diagnosis. These terms were chosen based on an initial screening performed by the researchers which suggested that these terms provided optimum sensitivity for procuring all relevant WHO literature for this review. Following the search of each term, the results will be expanded using a snowball strategy in order to ensure the inclusion of all relevant data. The terms that arise in the expanded search will be included in the final report.

Published Literature Search

Using the Ovid program, an electronic database search will be conducted on MEDLINE, EMBASE, Pubmed, and the Cochrane Library using the aforementioned search strategy. These searches will be filtered to only include studies with human subjects. An example of the search in MEDLINE can be found in Supplementary File 2.

Inclusion/Exclusion Criteria

Only fetuses with a structural congenital anomaly as listed in search string 1 will be included. Studies focusing on non-structural congenital anomalies will be excluded. Furthermore, all studies must focus on participants from LMICs; research focusing on high-income countries (HICs) will be excluded. Finally, studies that concentrate on antenatal diagnosis will be included, while studies focusing on postnatal diagnosis will be excluded. Included studies will be limited to the English language.

Study Design

No filters will be applied to study types; thus, all forms of evidence-based research will be included. This includes, but is not limited to, systematic reviews and meta-analyses, randomized controlled trials, prospective and retrospective cohort studies, case-control studies, and case series/reports. Qualitative studies and WHO/government policy documents and guidelines will be included to provide insight into the current practices regarding the provision of antenatal ultrasound and to help contextualize the use of antenatal ultrasound in LMICs.

Methodological Quality

To assess the methodological quality of the studies, the researchers will use the Cochrane Risk of Bias for Non-Randomized Studies of Intervention (ROBINS-I) and the revised tool to assess Risk of Bias in randomized trials (RoB 2.0) [13, 14]. These tools are widely used for critically appraising study methodology. For the purposes of this study, these tools will be used as a means of quality assessment, rather than as an inclusion/exclusion determinant. Two

https://mc.manuscriptcentral.com/bmjpo

Confidential: For Review Only

independent reviewers will score the articles according to the ROBINS-I and RoB 2.0 criteria and any discrepancies will be assessed by the research team until unanimity is reached.

Study Screening

References from the search results will be added to EndNote X8 and duplicates will be removed. The articles will then be uploaded to Covidence for the screening process. Two reviewers will screen the titles and abstracts in duplicate, removing any articles that do not meet the inclusion criteria. The remaining articles will be assessed by two reviewers independently in full-text and any remaining articles that do not adhere to the study criteria will be removed.

Data Extraction

Data extraction will be undertaken by the principal investigator. The following data will be extracted: place of study, study type, publication status, study population, patient cohort, gestational age at time of diagnosis, type of anomaly, type of ultrasound, percent receiving antenatal ultrasound, percent with any anomaly detected, percent with accurate antenatal diagnosis, reported sensitivity and specificity of antenatal ultrasound, training of ultrasound technician, referral rate for further antenatal care, referral rate to a tertiary pediatric surgery center, termination rate, mortality rate and complications as per the Clavien-Dindo classification. The following data will be collected regarding antenatal ultrasound policies: date, country/region, governing body, population coverage, intervention(s) and outcomes as detailed above.

Data Synthesis

Descriptive statistics and narrative synthesis will be utilized. The primary outcomes are the availability and effectiveness of ultrasound for the antenatal diagnosis of structural congenital anomalies in LMICs. The secondary outcomes are the effects of antenatal ultrasound diagnosis on neonatal morbidity and mortality, termination rates, and referral rates for further antenatal care. Data will be presented according to the following categories: availability of antenatal ultrasound, effectiveness of antenatal ultrasound, training of personnel performing ultrasound examination, secondary outcomes and antenatal ultrasound policies in LMICs. Information relating to the provision of antenatal ultrasound in isolation or as part of a perinatal care program will be noted, as will any details regarding private/government provisions of such services.

Based on the authors' experience, it is unlikely that a meta-analysis will be feasible due to limited availability of data. However, if there is appropriate data, a meta-analysis will be undertaken in duplicate by two independent authors and discrepancies resolved amongst the wider authorship. Appropriate data will be defined as: two or more sufficiently homogeneous studies comparing morbidity and/ or mortality between one group who has received antenatal ultrasound and another who has not. In addition, a meta-analysis may be undertaken if two or

https://mc.manuscriptcentral.com/bmjpo
Confidential: For Review Only

more studies have compared an intervention to improve antenatal ultrasound coverage or detection rates in a population. Meta-analysis will be undertaken in Stata and results presented using a forest plot. If there are over 10 studies in the meta-analysis a funnel plot will be undertaken to assess publication bias and a Galbraith plot to investigate heterogeneity in effect sizes. The quality of evidence will be assessed following GRADE guidelines.

Limitations

It is beyond the feasibility of this study to include articles in languages other than English. The researchers acknowledge this is a limitation. In order to aid interpretation of the systematic review findings, the number of studies included and excluded due to non-English language will be reported and depicted geographically in the final report.

Furthermore, it is important to note that antenatal ultrasound has further diagnostic capabilities, such as detecting abnormal growth or improper placental position. This systematic review will only focus on the detection of structural congenital anomalies, however, it may be prudent to consider other uses of antenatal ultrasound in further investigations.

DISCUSSION

The discrepancy in mortality due to congenital anomalies between HICs and LMICs is quite substantial. For instance, the survival rate for infants with gastroschisis in HICs is above 95%, while in many LMICs, there are few survivors of the condition [10]. This study aims to investigate the use of antenatal ultrasound to diagnose congenital anomalies in LMICs. Further, this study will examine the current policies and programs in place for antenatal ultrasound. Gaining a better understanding of the current policies and practices which increase the antenatal diagnosis of structural congenital anomalies may help to determine the most effective standards of practice. Increasing the early diagnosis of structural congenital anomalies may help to reduce the morbidity and mortality rates of congenital anomalies in LMICs. Many complications that arise from certain structural congenital anomalies could be avoided if the condition is detected antenatally and steps are taken to ensure safe delivery, such as planning the birth at a tertiary healthcare facility. This study may provide crucial information regarding the effects of antenatal ultrasound on morbidity and mortality rates from congenital anomalies in LMICs. This in turn can inform future studies aimed at improving availability and quality of antenatal ultrasound in LMICs to ultimately improve the health outcomes of infants born with these conditions.

ETHICS AND DISSEMINATION

The researchers of this study will be conducting a secondary analysis. No new data will be collected and there will be no direct interaction with participants. Therefore, it is not necessary to acquire ethical approval prior to conducting this review. Following data collection and

Confidential: For Review Only

analysis, the results will be submitted to a peer-reviewed scientific journal for publication. The results will also be shared in conferences pertaining to infant health, global health and global surgery.

Confidential: For Review Only

References

1. Apfeld, J.C., et al., *Infant, maternal, and geographic factors influencing gastroschisis related mortality in Zimbabwe.* Surgery, 2015. **158**(6): p. 1475-80.
2. Sitkin, N.A., et al., *Congenital anomalies in low- and middle-income countries: the unborn child of global surgery.* World J Surg, 2015. **39**(1): p. 36-40.
3. Collaborators, G.B.D.C.M., *Global, regional, national, and selected subnational levels of stillbirths, neonatal, infant, and under-5 mortality, 1980-2015: a systematic analysis for the Global Burden of Disease Study 2015.* Lancet, 2016. **388**(10053): p. 1725-1774.
4. Wu, V.K., D. Poenaru, and M.J. Poley, *Burden of surgical congenital anomalies in Kenya: a population-based study.* J Trop Pediatr, 2013. **59**(3): p. 195-202.
5. Ford, K., et al., *Gastroschisis: Bellwether for neonatal surgery capacity in low resource settings?* J Pediatr Surg, 2016. **51**(8): p. 1262-7.
6. Mashuda, F., et al., *Pattern and factors associated with congenital anomalies among young infants admitted at Bugando medical centre, Mwanza, Tanzania.* BMC Res Notes, 2014. **7**: p. 195.
7. *Congenital Anomalies*. 2016; Available from: http://www.who.int/mediacentre/factsheets/fs370/en/.
8. *What are the types of birth defects?* 2017; Available from: https://www.nichd.nih.gov/health/topics/birthdefects/conditioninfo/types.

9. Payne, N.R., et al., *Predicting the Outcome of Newborns with Gastroschisis.* Journal of pediatric surgery, 2009. **44**(5): p. 918-923.

10. Badrinath, R., et al., *Outcomes and unmet need for neonatal surgery in a resource-limited environment: estimates of global health disparities from Kampala, Uganda.* J Pediatr Surg, 2014. **49**(12): p. 1825-30.

11. Moher, D., et al., *Preferred reporting items for systematic review and meta-analysis protocols (PRISMA-P) 2015 statement.* Syst Rev, 2015. **4**: p. 1.

12. Shamseer, L., et al., *Preferred reporting items for systematic review and meta-analysis protocols (PRISMA-P) 2015: elaboration and explanation.* BMJ, 2015. **350**: p. g7647.

13. Higgins, J., et al., *Appraising the risk of bias in randomized trials using the Cochrane Risk of Bias Tool.* Cochrane Database of Systematic Reviews, 2016. **10**(Suppl 1).

14. Sterne, J.A., et al., *ROBINS-I: a tool for assessing risk of bias in non-randomised studies of interventions.* BMJ, 2016. **355**: p. i4919.

Confidential: For Review Only

Acknowledgements

We would like to thank the library services at King's College London for help with the systematic review process.

Contributors

NW conceived the idea for this study. All authors devised the study design. SG drafted the protocol with significant contributions from NW and AK. SG and SSB will perform the literature review and SG will draft the results.

Funding

NW receives funding from the Wellcome Trust to undertake a Clinical PhD in Global Health at King's Centre for Global Health and Health Partnerships, King's College London.

Competing Interests

The authors have no competing interests to declare.

Confidential: For Review Only

Supplementary File 1

Investigating the Use of Ultrasonography for the Antenatal Diagnosis of Structural Congenital Anomalies in Low- and Middle-Income Countries: A Systematic Review Protocol

PRISMA-P (Preferred Reporting Items for Systematic review and Meta-Analysis Protocols) 2015 Checklist

Section and topic	Item No	Checklist item	Section Where Addressed in Protocol
**Administrative information**			
Title:			
Identification	1a	Identify the report as a protocol of a systematic review	Title Page
Update	1b	If the protocol is for an update of a previous systematic review, identify as such	N/A
Registration	2	If registered, provide the name of the registry (such as PROSPERO) and registration number	Title Page
Authors:			
Contact	3a	Provide name, institutional affiliation, e-mail address of all protocol authors; provide physical mailing address of corresponding author	Title Page
Contributions	3b	Describe contributions of protocol authors and identify the guarantor of the review	Contributors
Amendments	4	If the protocol represents an amendment of a previously completed or published protocol, identify as such and list changes; otherwise, state plan for documenting important protocol amendments	Methods and Analysis
Support:			
Sources	5a	Indicate sources of financial or other support for the review	Funding
Sponsor	5b	Provide name for the review funder and/or sponsor	Funding
Role of sponsor or funder	5c	Describe roles of funder(s), sponsor(s), and/or institution(s), if any, in developing the protocol	Funding
**Introduction**			
Rationale	6	Describe the rationale for the review in the context of what is already known	Introduction
Objectives	7	Provide an explicit statement of the question(s) the review will address with reference to participants, interventions, comparators, and outcomes (PICO)	Aim; Objectives
**Methods**			
Eligibility criteria	8	Specify the study characteristics (such as PICO, study design, setting, time frame) and report characteristics (such as years considered, language, publication status) to be used as criteria for eligibility for the review	Search Strategy; Inclusion/Exclusion Criteria
Information sources	9	Describe all intended information sources (such as electronic databases, contact with study authors, trial registers or other grey literature sources) with planned dates of coverage	Published Literature Search

Confidential: For Review Only

Section and topic	Item No	Checklist item	Section Where Addressed in Protocol
Search strategy	10	Present draft of search strategy to be used for at least one electronic database, including planned limits, such that it could be repeated	Search Strategy; Table 1; Supplementary Document 2
Study records:			
Data management	11a	Describe the mechanism(s) that will be used to manage records and data throughout the review	Study Screening; Data Extraction
Selection process	11b	State the process that will be used for selecting studies (such as two independent reviewers) through each phase of the review (that is, screening, eligibility and inclusion in meta-analysis)	Study Screening
Data collection process	11c	Describe planned method of extracting data from reports (such as piloting forms, done independently, in duplicate), any processes for obtaining and confirming data from investigators	Data Extraction
Data items	12	List and define all variables for which data will be sought (such as PICO items, funding sources), any pre-planned data assumptions and simplifications	Data Extraction
Outcomes and prioritization	13	List and define all outcomes for which data will be sought, including prioritization of main and additional outcomes, with rationale	Data Extraction
Risk of bias in individual studies	14	Describe anticipated methods for assessing risk of bias of individual studies, including whether this will be done at the outcome or study level, or both; state how this information will be used in data synthesis	Methodological Quality
Data synthesis	15a	Describe criteria under which study data will be quantitatively synthesized	Data Synthesis
	15b	If data are appropriate for quantitative synthesis, describe planned summary measures, methods of handling data and methods of combining data from studies, including any planned exploration of consistency (such as I^2, Kendall's τ)	Data Synthesis
	15c	Describe any proposed additional analyses (such as sensitivity or subgroup analyses, meta-regression)	N/A
	15d	If quantitative synthesis is not appropriate, describe the type of summary planned	Data Synthesis
Meta-bias(es)	16	Specify any planned assessment of meta-bias(es) (such as publication bias across studies, selective reporting within studies)	Data Synthesis
Confidence in cumulative evidence	17	Describe how the strength of the body of evidence will be assessed (such as GRADE)	Data Synthesis

From: Shamseer L, Moher D, Clarke M, Ghersi D, Liberati A, Petticrew M, Shekelle P, Stewart L, PRISMA-P Group. Preferred reporting items for systematic review and meta-analysis protocols (PRISMA-P) 2015: elaboration and explanation. BMJ. 2015 Jan 2;349(jan02 1):g7647.

Confidential: For Review Only

Supplementary File 2

Investigating the Use of Ultrasonography for the Antenatal Diagnosis of Structural Congenital Anomalies in Low- and Middle-Income Countries: A Systematic Review Protocol

Database: **Ovid MEDLINE(R) Epub Ahead of Print, In-Process & Other Non-Indexed Citations, Ovid MEDLINE(R) Daily, Ovid MEDLINE and Versions(R)** <1946 to June 27, 2018>

Search Strategy:

No.	Search Terms	Results
1	exp Congenital Abnormalities/ or congenital anomal*.mp.	559922
2	exp Congenital Abnormalities/ or Congenital Abnormal*.mp.	555311
3	Congenital Malformation*.mp.	13826
4	Fetal Malformation*.mp.	1917
5	Birth Defect*.mp.	9122
6	Anencephaly.mp. or exp ANENCEPHALY/	3875
7	Conjoined Twins.mp. or exp Twins, Conjoined/	2022
8	exp Heart Defects, Congenital/ or Congenital Heart Defect*.mp.	142854
9	Anorectal Malformation*.mp.	1896
10	Anal Stenosis.mp.	389
11	Anal Atresia.mp. or exp Anus, Imperforate/	2815
12	Imperforate Anus.mp. or exp Anus, Imperforate/	2992
13	Biliary atresia.mp. or exp Biliary Atresia/	4482
14	exp Choledochal Cyst/ or Choledochal Cyst*.mp.	2825
15	exp Diaphragmatic Eventration/ or Diaphragmatic Eventration*.mp.	943
16	Esophageal Atresia.mp. or exp Esophageal Atresia/	4173
17	exp Tracheoesophageal Fistula/ or Tracheo-?esophageal fistula*.mp.	3676
18	Intestinal Atresia.mp. or exp Intestinal Atresia/	2301
19	Duodenal atresia.mp.	839
20	Colonic Atresia.mp.	152
21	Malrotation.mp.	2393
22	Apple peel syndrome.mp.	16
23	exp Hernias, Diaphragmatic, Congenital/ or congenital diaphragmatic hernia*.mp.	5530
24	Gastroschisis.mp. or exp GASTROSCHISIS/	2313
25	Abdominal Wall Defect*.mp.	2009
26	Exomphalos.mp. or exp Hernia, Umbilical/	3740
27	Omphalocele.mp. or exp Hernia, Umbilical/	4242
28	Congenital Limb Deformities.mp. or exp Limb Deformities, Congenital/	22483
29	exp Neural Tube Defects/ or Neural Tube Defect*.mp.	29051
30	exp Bronchogenic Cyst/ or Bronchogenic Cyst*.mp.	1810
31	exp Bronchopulmonary Sequestration/ or Bronchopulmonary sequestration*.mp.	2022
32	exp "Cystic Adenomatoid Malformation of Lung, Congenital"/ or Congenital Cystic adenomatoid malformation of lung*.mp.	874
33	Renal Anomal*.mp.	1185
34	exp Urogenital Abnormalities/ or Genito-urinary Anomalies.mp.	57735
35	exp Maxillofacial Abnormalities/ or Maxillofacial Abnormalit*.mp.	28265
36	exp Mouth Abnormalities/ or Mouth Abnormalit*.mp.	24459
37	Umbilical Hernia.mp. or exp Hernia, Umbilical/	4200
38	Hirschsprung disease.mp. or exp Hirschsprung Disease/	4567
39	exp Megacolon/ or Ganglionic Megacolon.mp.	7053
40	Rectosigmoid aganglionosis.mp.	15
41	Colonic Aganglionosis.mp.	311
42	Intestinal Aganglionosis.mp.	190
43	Volvulus.mp. or exp Intestinal Volvulus/	8927
44	LMICs.mp.	2001
45	(low- and middle-income countr*).mp. [mp=title, abstract, original title, name of substance word, subject heading word, floating sub-heading word, keyword heading word, protocol supplementary concept word, rare disease supplementary concept word, unique identifier, synonyms]	10910
46	Low-resource setting*.mp.	2719

Confidential: For Review Only

47	Underdeveloped Countries.mp.	817
48	low-income countries.mp.	4707
49	exp Developing Countries/ or Developing countr*.mp.	115302
50	middle-income countries.mp.	11136
51	limited resource setting*.mp.	253
52	Africa South of the Sahara.mp. or exp "Africa South of the Sahara"/	184102
53	Sub-Saharan Africa.mp. or exp "Africa South of the Sahara"/	190396
54	Less Resourced communities.mp.	1
55	Afghanistan.mp. or exp AFGHANISTAN/	6065
56	Albania.mp. or exp ALBANIA/	1182
57	Algeria.mp. or exp ALGERIA/	3621
58	American Samoa.mp.	354
59	Angola.mp. or exp ANGOLA/	1403
60	Argentina.mp. or exp ARGENTINA/	19610
61	Armenia.mp. or exp ARMENIA/	1676
62	Azerbaijan.mp. or exp AZERBAIJAN/	1765
63	Bangladesh.mp. or exp BANGLADESH/	12907
64	Belarus.mp. or exp "Republic of Belarus"/	2553
65	Belize.mp. or exp BELIZE/	868
66	Benin.mp. or exp BENIN/	3441
67	exp BHUTAN/ or Bhutan.mp.	628
68	Bolivia.mp. or exp BOLIVIA/	3594
69	(Bosnia and Herzegovina).mp. [mp=title, abstract, original title, name of substance word, subject heading word, floating sub-heading word, keyword heading word, protocol supplementary concept word, rare disease supplementary concept word, unique identifier, synonyms]	2477
70	Botswana.mp. or exp BOTSWANA/	2247
71	Brazil.mp. or exp BRAZIL/	101919
72	Bulgaria.mp. or exp BULGARIA/	7554
73	Burkina Faso.mp. or exp Burkina Faso/	3904
74	Burundi.mp. or exp BURUNDI/	871
75	Cabo Verde.mp. or exp Cabo Verde/	211
76	Cambodia.mp. or exp CAMBODIA/	4102
77	Cameroon.mp. or exp CAMEROON/	6804
78	Central African Republic.mp. or exp Central African Republic/	1096
79	Chad.mp. or exp CHAD/	1144
80	China.mp. or exp CHINA/	205799
81	Colombia.mp. or exp COLOMBIA/	12533
82	Comoros.mp. or exp COMOROS/	427
83	Democratic Republic of the Congo.mp. or exp "Democratic Republic of the Congo"/	4506
84	exp "Democratic Republic of the Congo"/ or DRC.mp.	4827
85	Republic of the Congo.mp. or exp Congo/	6176
86	Costa Rica.mp. or exp Costa Rica/	5088
87	Cote d'Ivoire.mp. or exp Cote d'Ivoire/	3527
88	Ivory Coast.mp. or exp Cote d'Ivoire/	3432
89	Croatia.mp. or exp CROATIA/	8261
90	Cuba.mp. or exp CUBA/	6343
91	Djibouti.mp. or exp DJIBOUTI/	372
92	Dominica.mp. or exp DOMINICA/	430
93	Dominican Republic.mp. or exp Dominican Republic/	2128
94	Ecuador.mp. or exp ECUADOR/	4722
95	exp EGYPT/ or Egypt.mp.	18702
96	El Salvador.mp. or exp El Salvador/	1319
97	Equatorial Guinea.mp. or exp Equatorial Guinea/	419
98	Eritrea.mp. or exp ERITREA/	518
99	Ethiopia.mp. or exp ETHIOPIA/	13354
100	Fiji.mp. or exp FIJI/	1620
101	Gabon.mp. or exp GABON/	1925
102	exp GAMBIA/ or Gambia.mp.	2958
103	exp "GEORGIA (REPUBLIC)"/ or exp GEORGIA/ or Georgia.mp.	15842
104	Ghana.mp. or exp GHANA/	9432
105	Grenada.mp. or exp GRENADA/	308
106	Guatemala.mp. or exp GUATEMALA/	3907
107	Guinea.mp. or exp GUINEA-BISSAU/ or exp EQUATORIAL GUINEA/ or exp GUINEA/ or exp PAPUA NEW GUINEA/ or exp NEW GUINEA/	160074
108	Guyana.mp. or exp GUYANA/	1136
109	Haiti.mp. or exp HAITI/	3707
110	Honduras.mp. or exp HONDURAS/	1736

Confidential: For Review Only

111	India.mp. or exp INDIA/	129215
112	Indonesia.mp. or exp INDONESIA/	13107
113	exp Iran/ or Islamic Republic of Iran.mp.	21280
114	exp IRAQ/ or Iraq.mp.	9035
115	Jamaica.mp. or exp JAMAICA/	4427
116	Jordan.mp. or exp JORDAN/	6040
117	Kazakhstan.mp. or exp KAZAKHSTAN/	3153
118	Kenya.mp. or exp KENYA/	18798
119	Kiribati.mp. or exp Micronesia/	1934
120	Democratic People's Republic of Korea.mp. or exp "Democratic People's Republic of Korea"/	246
121	Kosovo.mp. or exp KOSOVO/	840
122	Kyrgyz Republic.mp. or exp Kyrgyzstan/	1225
123	exp Laos/ or Lao PDR.mp.	1876
124	Laos.mp. or exp LAOS/	2431
125	Lebanon.mp. or exp LEBANON/	5011
126	Lesotho.mp. or exp LESOTHO/	645
127	Liberia.mp. or exp LIBERIA/	1667
128	Libya.mp. or exp LIBYA/	1554
129	Macedonia Republic.mp. or exp "Macedonia (Republic)"/	475
130	Madagascar.mp. or exp MADAGASCAR/	4734
131	Malawi.mp. or exp MALAWI/	6317
132	Malaysia.mp. or exp MALAYSIA/	18390
133	Maldives.mp. or exp Indian Ocean Islands/	10595
134	Mali.mp. or exp MALI/	3511
135	Marshall Islands.mp. or exp Micronesia/	1959
136	Mauritania.mp. or exp MAURITANIA/	626
137	Mauritius.mp. or exp MAURITIUS/	953
138	exp MEXICO/ or Mexico.mp.	53282
139	Micronesia.mp. or exp MICRONESIA/	2243
140	Moldova.mp. or exp MOLDOVA/	920
141	Mongolia.mp. or exp MONGOLIA/	3809
142	Montenegro.mp. or exp MONTENEGRO/	749
143	Morocco.mp. or exp MOROCCO/	6855
144	Mozambique.mp. or exp MOZAMBIQUE/	3236
145	Myanmar.mp. or exp MYANMAR/	3198
146	Namibia.mp. or exp NAMIBIA	1502
147	Nauru.mp. or exp Micronesia/	1921
148	Nepal.mp. or exp NEPAL/	9337
149	Nicaragua.mp. or exp NICARAGUA/	1949
150	exp NIGER/ or Niger.mp.	12299
151	Nigeria.mp. or exp NIGERIA/	32627
152	Pakistan.mp. or exp PAKISTAN/	20153
153	exp PANAMA/ or Panama.mp.	4265
154	Papua New Guinea.mp. or exp Papua New Guinea/	5003
155	Paraguay.mp. or exp PARAGUAY/	1433
156	Peru.mp. or exp PERU/	11249
157	Philippines.mp. or exp PHILIPPINES/	10698
158	Romania.mp. or exp ROMANIA/	11521
159	exp Russia/ or Russian Federation.mp.	51376
160	Rwanda.mp. or exp RWANDA/	2883
161	exp AMERICAN SAMOA/ or Samoa.mp. or exp SAMOA/ or exp "INDEPENDENT STATE OF SAMOA"/	1024
162	(Sao Tome and Principe).mp. [mp=title, abstract, original title, name of substance word, subject heading word, floating sub-heading word, keyword heading word, protocol supplementary concept word, rare disease supplementary concept word, unique identifier, synonyms]	135
163	Senegal.mp. or exp SENEGAL/	7067
164	Serbia.mp. or exp SERBIA/	4574
165	Sierra Leone.mp. or exp Sierra Leone/	2103
166	Solomon Islands.mp. or exp Melanesia/	6193
167	Somalia.mp. or exp SOMALIA/	1968
168	Somaliland.mp.	136
169	South Africa.mp. or exp South Africa/	47075
170	South Sudan.mp. or exp South Sudan/	397
171	Sri Lanka.mp. or exp Sri Lanka/	7342
172	Saint Lucia.mp. or exp Saint Lucia/	114
173	(Saint Vincent and the Grenadines).mp. [mp=title, abstract, original title, name of substance word, subject heading word, floating sub-heading word, keyword heading word, protocol supplementary concept word, rare disease supplementary concept word, unique identifier, synonyms]	57

For peer review only

174	exp SUDAN/ or Sudan.mp.	8429
175	Suriname.mp. or exp SURINAME/	1098
176	Swaziland.mp. or exp SWAZILAND/	818
177	Syrian Arab Republic.mp.	73
178	Syria.mp. or exp SYRIA/	2407
179	Tajikistan.mp. or exp TAJIKISTAN/	910
180	Tanzania.mp. or exp TANZANIA/	12921
181	Thailand.mp. or exp THAILAND/	32186
182	exp Timor-Leste/ or Timor-Leste.mp.	311
183	East Timor.mp.	187
184	Togo.mp. or exp TOGO/	1491
185	Tonga.mp. or exp TONGA/	463
186	Tunisia.mp. or exp TUNISIA/	9057
187	exp TURKEY/ or Turkey.mp.	46898
188	Turkmenistan.mp. or exp TURKMENISTAN/	717
189	Tuvalu.mp. or exp Micronesia/	1884
190	Uganda.mp. or exp UGANDA/	13956
191	Ukraine.mp. or exp UKRAINE/	16702
192	Uzbekistan.mp. or exp UZBEKISTAN/	2161
193	Vanuatu.mp. or exp VANUATU/	629
194	Venezuela.mp. or exp VENEZUELA/	6620
195	exp VIETNAM/ or Vietnam.mp.	15919
196	(West Bank and Gaza).mp. [mp=title, abstract, original title, name of substance word, subject heading word, floating sub-heading word, keyword heading word, protocol supplementary concept word, rare disease supplementary concept word, unique identifier, synonyms]	194
197	Republic of Yemen.mp. or exp Yemen/	1264
198	Zambia.mp. or exp ZAMBIA/	5404
199	Zimbabwe.mp. or exp ZIMBABWE/	6879
200	Antenatal Diagnosis.mp.	2383
201	Prenatal Diagnosis.mp. or exp Prenatal Diagnosis/	74636
202	Antenatal Screening.mp.	1193
203	Prenatal Screening.mp.	2579
204	Antenatal Ultrasound.mp.	702
205	Prenatal Ultrasound.mp.	2203
206	Antenatal Ultrasonography.mp.	200
207	Prenatal Ultrasonography.mp. or exp Ultrasonography, Prenatal/	30427
208	1 or 2 or 3 or 4 or 5 or 6 or 7 or 8 or 9 or 10 or 11 or 12 or 13 or 14 or 15 or 16 or 17 or 18 or 19 or 20 or 21 or 22 or 23 or 24 or 25 or 26 or 27 or 28 or 29 or 30 or 31 or 32 or 33 or 34 or 35 or 36 or 37 or 38 or 39 or 40 or 41 or 42 or 43	607001
209	44 or 45 or 46 or 47 or 48 or 49 or 50 or 51 or 52 or 53 or 54 or 55 or 56 or 57 or 58 or 59 or 60 or 61 or 62 or 63 or 64 or 65 or 66 or 67 or 68 or 69 or 70 or 71 or 72 or 73 or 74 or 75 or 76 or 77 or 78 or 79 or 80 or 81 or 82 or 83 or 84 or 85 or 86 or 87 or 88 or 89 or 90 or 91 or 92 or 93 or 94 or 95 or 96 or 97 or 98 or 99 or 100 or 101 or 102 or 103 or 104 or 105 or 106 or 107 or 108 or 109 or 110 or 111 or 112 or 113 or 114 or 115 or 116 or 117 or 118 or 119 or 120 or 121 or 122 or 123 or 124 or 125 or 126 or 127 or 128 or 129 or 130 or 131 or 132 or 133 or 134 or 135 or 136 or 137 or 138 or 139 or 140 or 141 or 142 or 143 or 144 or 145 or 146 or 147 or 148 or 149 or 150 or 151 or 152 or 153 or 154 or 155 or 156 or 157 or 158 or 159 or 160 or 161 or 162 or 163 or 164 or 165 or 166 or 167 or 168 or 169 or 170 or 171 or 172 or 173 or 174 or 175 or 176 or 177 or 178 or 179 or 180 or 181 or 182 or 183 or 184 or 185 or 186 or 187 or 188 or 189 or 190 or 191 or 192 or 193 or 194 or 195 or 196 or 197 or 198 or 199 (1385344)	1385344
210	200 or 201 or 202 or 203 or 204 or 205 or 206 or 207	77525
211	208 and 209 and 210	896

Confidential: For Review Only
